# Combination immunotherapy for colorectal cancer: Clinical applications, rationale, challenges, and future perspectives

**DOI:** 10.1016/j.xcrm.2026.102728

**Published:** 2026-04-07

**Authors:** Jiang Fei, Changjing Cai, Wantao Wu, Hong Shen, Ying Han, Shan Zeng

**Affiliations:** 1 Department of Oncology, Xiangya Hospital, Central South University, Changsha, Hunan 410008, China; 2 National Clinical Research Center for Geriatric Disease (Xiangya Hospital), Central South University, Changsha, Hunan 410008, P.R. China

**Keywords:** colorectal cancer, immunotherapy, combination therapy, TME, ICIs

## Abstract

Colorectal cancer (CRC) is a leading cause of cancer-related mortality. Although immunotherapy has demonstrated remarkable efficacy in deficient mismatch repair (dMMR)/microsatellite instability-high (MSI-H) tumors, most proficient mismatch repair (pMMR)/microsatellite stability (MSS)/microsatellite instability-low (MSI-L) patients derive limited benefit. Combination strategies—including immune checkpoint inhibitors (ICIs), chemotherapy, targeted therapy, and radiotherapy—offer promise for overcoming resistance. This review examines recent clinical advances in combination immunotherapy for CRC, with a focus on tumor microenvironment (TME) modulation and predictive biomarkers. By bridging preclinical insights and clinical applications, we aim to facilitate the optimization of therapeutic strategies and extend the benefits of immunotherapy across CRC subtypes.

## Graphical abstract

**Figure:**
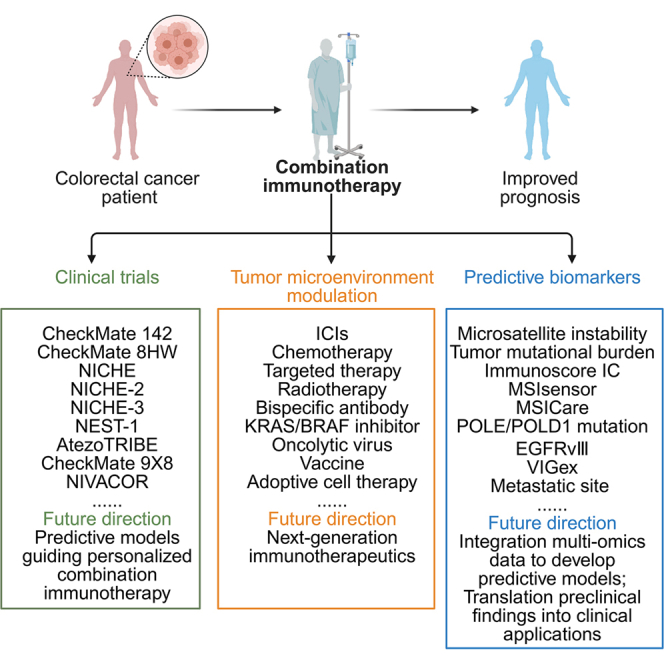

## Teaser

Fei et al. review combination immunotherapy strategies for colorectal cancer, focusing on overcoming resistance in pMMR/MSS/MSI-L tumors. They highlight integrating ICIs with chemotherapy, targeted therapy, and radiotherapy and discuss insights into the tumor microenvironment and biomarkers to improve outcomes.

## Introduction

Colorectal cancer (CRC) is the third most common malignancy worldwide and accounts for nearly 10% of all cancer-related deaths.^1^ Although the overall 5-year survival rate for CRC is approximately 64%, this rate drops sharply to below 15% in metastatic CRC (mCRC).^2^ Molecularly, CRC is categorized into two main subtypes: deficient mismatch repair (dMMR)/microsatellite instability-high (MSI-H) and proficient mismatch repair (pMMR)/microsatellite stability (MSS)/microsatellite instability-low (MSI-L). MMR status is typically assessed via immunohistochemical (IHC) of MMR proteins (Mut L homologue 1 [MLH1]; post-meiotic segregation 2 [PMS2]; Mut S homologue 2 [MSH2]; and Mut S homologue 6 [MSH6]), polymerase chain reaction (PCR), or next-generation sequencing (NGS).^3^ Standard treatments, including surgery, chemotherapy, and targeted therapy, often yield suboptimal outcomes,^4^ driving interest in immunotherapy. Immunotherapy, particularly immune checkpoint inhibitors (ICIs), have shown remarkable efficacy in dMMR/MSI-H CRC.^5^

According to 2025 colon cancer and rectal cancer guidelines of national comprehensive cancer network (NCCN), 1. For non-metastatic dMMR/MSI-H or polymerase epsilon/polymerase delta 1 (POLE/POLD1) mutation with ultra-hypermutated phenotype colon cancer: (1) resectable clinical T4b or bulky nodal disease: neoadjuvant checkpoint inhibitor immunotherapy can be considered, followed by colectomy with *en bloc* removal of regional lymph nodes; (2) locally unresectable or medically inoperable: checkpoint inhibitor immunotherapy can be considered, followed by evaluating for complete response (CR) or conversion to resectability; (3) adjuvant treatment: FOLFOX (fluorouracil, calcium folinate, and oxaliplatin)/CAPEOX (capecitabine and oxaliplatin) with atezolizumab can be considered for stage ⅡC/Ⅲ, and fluoropyrimidine therapy with atezolizumab is a category 2B recommendation for stage ⅡC (T4bN0M0). 2. For suspected or proven dMMR/MSI-H (or POLE/POLD1 mutation with ultra-hypermutated phenotype) metastatic synchronous colon and rectal adenocarcinoma (any T, any N, M1) with resectable synchronous liver-only and/or lung-only metastases, checkpoint inhibitor immunotherapy can be considered, followed by synchronous or staged colectomy (or resection of rectal lesion) and resection and/or local therapy of metastatic disease. 3. dMMR/MSI-H or POLE/POLD1 mutation with ultra-hypermutated phenotype CRC with resectable metachronous metastases, if no previous immunotherapy, checkpoint inhibitor immunotherapy can be considered as initial treatment, followed by observation or resection and/or local therapy. 4. Systemic therapy for advanced or metastatic dMMR/MSI-H or POLE/POLD1 mutation with ultra-hypermutated phenotype CRC: (1) candidate for immunotherapy and no prior immunotherapy received, and checkpoint inhibitor immunotherapy can be considered (patients should be followed-up closely for 10 weeks to assess for response), followed by re-evaluation of disease status every 2–3 months; (2) if checkpoint inhibitor monotherapy was previously received, nivolumab plus ipilimumab can be considered. 5. dMMR/MSI-H or POLE/POLD1 mutation with ultra-hypermutated phenotype (T3, N any; T1-2, N1-2; T4, N any, locally unresectable or medically inoperable) rectal cancer (if no previous treatment with a checkpoint inhibitor): checkpoint inhibitor immunotherapy can be considered as neoadjuvant/definitive treatment, followed by re-evaluating disease status every 2–3 months.^6^^,^^7^ However, the KEYNOTE-177 trial reported that pembrolizumab monotherapy did not confer an overall survival (OS) advantage over chemotherapy as first-line treatment in dMMR/MSI-H mCRC.^8^ Moreover, most pMMR/MSS tumors remain refractory to immunotherapy.^9^ Thus, identifying optimal combination immunotherapeutic strategies for CRC represents a critical unmet need.

The tumor microenvironment (TME) plays a pivotal role in CRC progression and therapeutic response. Although TME composition varies across tumor types, it generally consists of immune cells, stromal cells, vasculature, and the extracellular matrix (ECM).^10^ CRC is further stratified into four consensus molecular subtypes (CMSs): CMS1 (microsatellite instability immune, 14%), characterized by hypermutated, microsatellite unstable with strong immune activation; CMS2 (canonical, 37%), marked by epithelial tumors with activated Wnt and Myc signaling pathways; CMS3 (metabolic, 13%), featuring epithelial tumors with evident metabolic dysregulation; and CMS4 (mesenchymal, 23%), characterized by prominent transforming growth factor beta (TGF-β) activation, stromal invasion, and angiogenesis. CMS 2–4 subtypes are largely pMMR/MSS and generally exhibit less immunogenic, immunosuppressive TMEs.^11^ Compared to dMMR/MSI-H tumors, pMMR/MSS tumors typically display lower immune infiltration and immune checkpoint expression. Furthermore, immunogenicity varies across metastatic sites: liver and peritoneal metastases tend to be more immunosuppressive than pulmonary metastases.^12^ These observations suggest that combination therapies may improve immunotherapy outcomes by reprogramming the TME, although the TME landscape under combination immunotherapy in CRC remains incompletely characterized.

In this review, we summarize recent advances in combination immunotherapy for CRC from both clinical and tumor immune microenvironment perspectives. We survey clinical trials of ICIs and explore combination modalities such as dual checkpoint blockade, chemotherapy, targeted therapy, radiotherapy, and other emerging strategies. Additionally, we discuss how combination therapies reshape the TME and examine predictive biomarkers and clinical parameters of treatment response, drawing on insights from basic and translational studies. Ultimately, we aim to bridge clinical observations and mechanistic understanding to help identify the most effective immunotherapy combinations for CRC.

## Immunotherapy combination strategies for dMMR/MSI-H CRC

### First-line therapy

The CheckMate 142 trial established strong and sustained clinical benefit with nivolumab plus low-dose ipilimumab, supporting its use as a first-line treatment for mCRC.^13^ CheckMate 8HW further corroborated these findings, demonstrating superior progression-free survival (PFS) and health-related quality of life (HRQoL) with nivolumab plus ipilimumab compared to chemotherapy^14^ (Table 1). A prespecified interim analysis of this trial also showed that nivolumab plus ipilimumab led to longer PFS than nivolumab alone across all treatment lines.^15^ Together, these results support this dual immunotherapy regimen as a standard of care (SOC) in mCRC. Beyond metastatic disease, short-course nivolumab plus ipilimumab has shown high efficacy in locally advanced rectal cancer (LARC) and may represent a non-surgical alternative in selected cases.^16^ The ongoing COMMIT trial (NCT02997228) is evaluating atezolizumab combined with FOLFOX and bevacizumab, which may further expand first-line options in the future.

**Table 1:** Immunotherapy combination strategies for CRC

ICIs	Combination	Setting	Phase	Patients	Results	Trial number	Reference
**Part 1 Immunotherapy combination strategies for dMMR/MSI-H CRC**							

Nivolumab	ipilimumab	1L	2	MSI-H mCRC	ORR: 69% DCR: 84%	NCT02060188	Lenz et al.^13^
Nivolumab	ipilimumab	1L	1	MSI-H LARC	cCR: 100% (5/5)	EU2022-500646-14-00	Pfeiffer et al.^16^
Nivolumab	ipilimumab	Neoadjuvant	2	early-stage MSI-H CC	pCR: 69%	NCT03026140	Verschoor et al.^17^
Nivolumab	ipilimumab	Neoadjuvant	3	MSI-H LACC	pCR: 67%	NL58483.031.16	Chalabi et al.^18^
Nivolumab	relatlimab	Neoadjuvant	2	MSI-H LACC	pCR: 68% MPR: 91%	NCT03026140	Gooyer et al.^19^
Toripalimab	celecoxib	Neoadjuvant	2	MSI-H LACC	pCR: 87%	NCT03926338	Hu et al.^20^
Atezolizumab	SCRT bevacizumab	Neoadjuvant	2	non-advanced MSI-H RC	OP rate: 100% (6/6)	NCT04017455	Gooyer et al.^21^
Sintilimab	IBI310	Neoadjuvant	1b	MSI-H LACRC	pCR: 80%	NCT05890742	Xu et al.^22^
Atezolizumab	mFOLFOX	Adjuvant	3	stage Ⅲ dMMR CC	3-year DFS: 86.4%	NCT02912559	Sinicrope et al.^23^
Nivolumab	ipilimumab	≥2L	2	MSI-H mCRC	ORR: 55% DCR: 80%	NCT02060188	Overman et al.^24^
Nivolumab	ipilimumab	≥2L	2	MSI-H mCRC	5-year iPFS rates: 61.3% 5-year OS rates: 71.1%	NCT03350126	Depotte et al.^25^
Pembrolizumab	SABR	–	–	MSI-H mCRC	ORR: 29% DCR: 50%	–	Gandini et al.^26^

**Part 2 Dual immune checkpoint inhibition for pMMR/MSS/MSI-L CRC**							

Nivolumab	ipilimumab celecoxib	Neoadjuvant	2	early-stage MSS CC	pCR: 10%	NCT03026140	Verschoor et al.^17^
Durvalumab	tremelimumab	Neoadjuvant	1	MSS CRC	mRFS: 9.7 months OS: 24.5 months	NCT02754856	Kanikarla Marie et al.^27^
Balstilimab	botensilimab	Neoadjuvant	2	MSS CRC	pCR: 31.8% MPR: 40.9%	NCT05571293	Hissong et al.^28^
Balstilimab	botensilimab	≥2L	1a/1b	MSS mCRC	ORR: 22% DCR: 73%	NCT03860272	El-Khoueiry et al.^29^
Pembrolizumab	muzastotug	3L	1b/2	MSS mCRC	ORR: 26% mPFS: 6.7 months	NCT05405595	Li et al.^30^
Atezolizumab	vilastobart	≥2L	2	MSS mCRC	ORR: 27% (NLM) 29% (LM)	NCT04896697	Fakih et al.^31^

**Part 3 Combination with chemotherapy regardless of MMR or MSI status**							

Atezolizumab	FOLFOXIRI bevacizumab	1L	2	mCRC	mPFS: 13.1 months	NCT03721653	Antoniotti et al.^32^
Nivolumab	mFOLFOX bevacizumab	1L	2	mCRC	PFS: 11.9 months ORR: 60%	NCT03414983	Lenz et al.^33^
Nivolumab	FOLFOXIRI bevacizumab	1L	2	RAS/BRAF^MT^ advanced CRC	ORR: 78.9% DCR: 96.2%	NCT04072198	Damato et al.^34^
Cadonilimab	mFOLFOXIRI	Neoadjuvant	2	LACRC	pCR: 26.8% MPR: 68.3%	NCT05571644	Zhang et al.^35^
Camrelizumab	irinotecan/surufatinib GM-CSF	2L	1b/2	advanced CRC	ORR: 30.0% DCR: 93.3%	NCT04929652	Li et al.^36^
Sintilimab	HAIC regorafenib	Adjuvant	2	post-hepatectomy	1-year RFS rate: 67.8%	NCT05753163	Wang et al.^37^

**Part 4 Combination with chemotherapy for pMMR/MSS/MSI-L CRC**							

Durvalumab Tremelimumab	mFOLFOX	1L	1b/2	RAS^MT^ MSS mCRC	3-month PFS rate: 90.7% ORR: 64.5%	NCT03202758	Thibaudin et al.^38^
Tislelizumab	CAPEOX	Neoadjuvant	2	MSS LACC	pCR: 51.85%	NCT06124378	Lin et al.^39^
Cadonilimab	CAPEOX	Neoadjuvant	2	MSS LARC	pCR: 15.4%	NCT05815303	Zhou et al.^40^
Anti-PD-1	rectal arterial infusion chemotherapy	Neoadjuvant	2	MSS LARC	pCR: 35.5%	NCT05307198	Li et al.^41^
Nivolumab	trifluridine/tipiracil	≥3L	2	MSS mCRC	mPFS: 2.8 months	NCT02860546	Patel et al.^42^
Pembrolizumab	azacitidine	≥2L	2	MSS mCRC	ORR: 3%	NCT02260440	Kuang et al.^43^
Nivolumab Ipilimumab	temozolomide	≥2L	2	MGMT-silenced MSS mCRC	ORR: 45%; mPFS: 7.0 months mOS: 18.4 months	NCT03832621	Morano et al.^44^
Sintilimab	chidamide/bevacizumab	≥3L	2	MSS mCRC	mPFS: 7.3 months	NCT04724239	Wang et al.^45^

**Part 5 Combination with targeted therapy for pMMR/MSS/MSI-L CRC**							

Avelumab	cetuximab	3L	2	RAS^WT^ MSS mCRC	mOS: 11.6 months mPFS: 3.6 months	NCT04561336	Martinelli et al.^46^
Nivolumab Ipilimumab	panitumumab	≥2L	2	RAS/BRAF^WT^ MSS mCRC	ORR (12-week): 35% mPFS: 5.7 months	NCT03442569	Lee et al.^47^
Sintilimab	anlotinib	1L	2	advanced mCRC	ORR: 48.3% DCR: 89.7%	NCT04271813	Wang et al.^48^
Pembrolizumab	lenvatinib	≥2L	3	MSS mCRC	ORR: 11.9% mOS: 13.4 months	NCT04776148	Xu et al.^49^
Nivolumab Ipilimumab	regorafenib	2L	1	MSS mCRC	ORR: 36.4% OS: >22 months	NCT04362839	Xiao et al.^50^
Durvalumab	cabozantinib	≥3L	2	advanced MSS mCRC	ORR: 27.6% DCR: 86.2%	NCT03539822	Saeed et al.^51^
Nivolumab	regorafenib	≥3L	1b	advanced MSS CRC	ORR: 36% mPFS: 7.9 months	NCT03406871	Fukuoka et al.^52^
Pembrolizumab	lenvatinib	≥2L	2	advanced MSS CRC	ORR: 22% DCR: 47%	NCT03797326	Gomez-Roca et al.^53^
Spartalizumab	trametinib dabrafenib	≥1L	2	BRAF^V600E^ MSS mCRC	ORR: 25%	NCT03668431	Tian et al.^54^
Durvalumab	trametinib	≥2L	2	MSS mCRC	ORR: 3.4% mPFS: 3.2 months	NCT03428126	Johnson et al.^55^
Atezolizumab	cobimetinib	3L	3	MSS mCRC	mOS: 8.87 months	NCT02788279	Eng et al.^56^

**Part 6 Combination with chemotherapy and targeted therapy for pMMR/MSS/MSI-L CRC**							

Sintilimab	chemotherapy cetuximab	1L	1b/2	RAS/BRAF^MT^ MSS mCRC	ORR: 86.7% DCR: 100.0%	NCT06776757	Wang et al.^57^
Sintilimab	CAPEOX bevacizumab	1L	2	RAS^MT^ MSS mCRC	ORR: 84.0% DCR: 100.0%	NCT05171660	Zhu et al.^58^
Pembrolizumab	CAPEOX bevacizumab	1L	2	MSS mCRC	ORR: 75% DCR: 100%	NCT04262687	Tougeron et al.^59^
Serplulimab	CAPEOX HLX04	1L	2/3	MSS mCRC	mPFS: 17.2 months	NCT04547166	Wang et al.^60^
Camrelizumab	mFOLFOXIRI bevacizumab	Neoadjuvant	2	MSS LARC	CR: 44.8%	ChiCTR2100054182	Wang et al.^61^
Toripalimab	chemotherapy surufatinib	2L	2	RAS/BRAF^MT^ MSS mCRC	ORR: 31.25% DCR: 93.75%	NCT04653480	Zhang et al.^62^
Penpulimab	irinotecan anlotinib	2L	2	MSS mCRC	ORR: 42.9% DCR: 90.5%	NCT05229003	Wang et al.^63^
Envafolimab	irinotecan cetuximab	≥3L	2	RAS/BRAF^WT^ MSS mCRC	ORR: 50% DCR: 87.5%	NCT06321081	Zhang et al.^64^
Tislelizumab	irinotecan cetuximab	≥3L	2	RAS^WT^ MSS mCRC	ORR: 33% DCR: 79%	ChiCTR2000035642	Xu et al.^65^
Enlonstobart	irinotecan becotatug	≥3L	2	RAS/BRAF^WT^ non-dMMR/MSI-H	ORR: 44.1% DCR: 82.4%	NCT06089330	Xu et al.^66^
Avelumab	irinotecan cetuximab	≥2L	2	RAS^WT^ MSS mCRC	ORR: 21.4%	NCT03608046	van den Eynde et al.^67^
Atezolizumab	capecitabine bevacizumab	≥2L	2	MSS mCRC	mPFS: 4.4 months	NCT02873195	Mettu et al.^68^

**Part 7 Combination with radiotherapy for pMMR/MSS/MSI-L CRC**							

Pembrolizumab	SBRT	≥2L	1b	MSS mCRC	1-year RFS rate: 67%	NCT02837263	Deming et al.^69^
Atezolizumab	SBRT	≥2L	2	mCRC	mPFS: 1.4 months	NCT02992912	Levy et al.^70^
Nivolumab Ipilimumab	radiotherapy	≥3L	2	MSS mCRC	ORR: 7.5% DCR: 17.5%	NCT03104439	Parikh et al.^71^
Durvalumab Tremelimumab	radiotherapy	≥2L	2	MSS mCRC	ORR: 8.3%	NCT03122509	Segal et al.^72^

**Part 8 Combination with radiotherapy and chemotherapy for pMMR/MSS/MSI-L LARC neoadjuvant therapy**							

Nivolumab	CRT	Neoadjuvant	2	MSS LARC	mpCR: 65%	NCT03921684	Brenner et al.^73^
Tislelizumab	CRT	Neoadjuvant	2	MSS LARC	pCR: 23%	NCT05245474	Pang et al.^74^
Toripalimab	SCRT CAPEOX	Neoadjuvant	2	MSS LARC	CR: 55.8%	NCT04518280	Wang et al.^75^
Tislelizumab	HFRT CAPEOX	Neoadjuvant	2	LARC	ORR: 100% pCR: 56.5%	NCT05176964	Zheng et al.^76^
Toripalimab	HFRT chemotherapy	≥1L	2	LRCC	ORR: 82.9%/65.4%	NCT05628038	Wu et al.^77^
Serplulimab	LCRT FOLFOXIRI	Neoadjuvant	2	LALRC	CR: 75%	NCT06099951	Peng et al.^78^

**Part 9 Combination with radiotherapy and targeted therapy for pMMR/MSS/MSI-L CRC**							

Toripalimab	SCRT fruquintinib	Neoadjuvant	2	MSS LARC	pCR: 37.5%	NCT05763927.	Chen et al.^79^
Atezolizumab	SCRT Bevacizumab	Neoadjuvant	2	non-advanced MSS RC	OP rate: 42%	NCT04017455	Gooyer et al.^21^
Tislelizumab	SABR fruquintinib	≥2L	2	mCRC	ORR: 34.3% DCR: 77.1%	NCT04948034	Chen et al.^80^
Tislelizumab	SBRT fruquintinib	≥2L	2	MSS mCRC	ORR: 26% DCR: 83%	NCT04924179	Yuan et al.^81^

**Part 10 Combination with chemotherapy, targeted therapy and radiotherapy for pMMR/MSS/MSI-L CRC**							

Tislelizumab	CAPEOX, SCRT bevacizumab/cetuximab	1L	–	unresectable MSS mRC	ORR: 82.4% DCR: 100%	–	Lin et al.^82^
Adebrelimab	CAPEOX, SCRT fruquintinib	Neoadjuvant	2	high-risk LARC	CR: 65%	NCT06234007	Lin et al.^83^
Sintilimab	CAPEOX, SCRT alotinib	Neoadjuvant	2	MSS LARC	CR: nearly 100%	ChiCTR2100054135	Zhao et al.^84^

**Part 11 Promising candidates for combination immunotherapies**							

Spartalizumab	sabatolimab	≥2L	1/1b	advanced CRC	2 patients responses	NCT02608268	Curigliano et al.^85^
Pembrolizumab	favezelimab	≥2L	3	CPS≥1 MSS CRC	mOS: 7.3 months	NCT05064059	Passhak et al.^86^
Ivonescimab	ligufalimab FOLFOXIRI	1L	2	MSS mCRC	ORR: 88.2% DCR: 100%	NCT05382442	Deng et al.^87^
IBI363	bevacizumab	≥2L	1a/1b	advanced Non-dMMR CRC	ORR: 23.5%	NCT05460767	Lin et al.^88^
Nivolumab	encorafenib cetuximab	≥2L	1/2	BRAF^V600E^ MSS mCRC	ORR: 45% DCR: 95%	NCT04017650	Morris et al.^89^
Durvalumab	olaparib cediranib	≥2L	2	MSS mCRC	SD: 10%	NCT03851614	Hernando-Calvo et al.^90^
Atezolizumab	chemotherapy pelareorep	3L	1/2	MSS mCRC	SD: 33.3%	EudraCT2020-003996-16	Ungerechts et al.^91^
Durvalumab Tremelimumab	PexaVec	≥2L	1/2	MSS mCRC	mPFS: 2.3 months	NCT03206073	Monge et al.^92^
Pembrolizumab	Nous-209	1L	1/2	advanced/metastatic MSI-H CRC	ORR: 71%	NCT04041310	Overman et al.^93^
Nivolumab Ipilimumab	vaccine	1L	2	MSS mCRC	improved PFS (HR = 0.73)	NCT05141721	Hecht et al.^94^
Atezolizumab	polyPEPI1018	≥2L	2	MSS mCRC	no objective responses	NCT05243862	Hubbard et al.^95^
ACT	CAPEOX bevacizumab	1L	3	mCRC	mPFS: 14.8 months mOS: not reached	NCT03950154	Pan et al.^96^
Durvalumab	tazemetostat	≥2L	2	advanced MSS CRC	DCR: 35.3%	NCT04705818	Palmieri et al.^97^
Balstilimab	CR6068	≥2L	1/2	MSS mCRC	ORR: 11% DCR: 50%	NCT05205330	Pietrantonio et al.^98^
Tislelizumab	fruquintinib FMT	≥3L	2	MSS mCRC	mPFS: 9.6 months mOS: 13.7 months	ChiCTR2100046768	Zhao et al.^99^

ACT, adoptive cell therapy; CAPEOX, capecitabine and oxaliplatin; CC, colon cancer; cCR, clinical complete response; CPS, combined positive score; CRC, colorectal cancer; CRT, chemoradiotherapy; DCR, disease control rate; dMMR, deficient mismatch repair; FMT, fecal microbiota transplantation; FOLFIRI, fluorouracil, calcium folinate and irinotecan; FOLFOX, fluorouracil, calcium folinate and oxaliplatin; FOLFOXIRI, fluorouracil, calcium folinate, oxaliplatin and irinotecan; GM-CSF, granulocyte-macrophage colony-stimulating factor; HAIC, hepatic arterial infusion chemotherapy; HFRT, hypofraction radiotherapy; HR, hazard ratio; ICIs, immune checkpoint inhibitors; iPFS, immune response evaluation criteria in solid tumors (RECIST) PFS; LACRC, locally advanced colorectal cancer; LACC, locally advanced colon cancer; LALRC, locally advanced low rectal cancer; LARC, locally advanced rectal cancer; LCRT, long-course radiotherapy; LM, liver metastases; mCRC, metastatic colorectal cancer; MGMT, O6-methylguanine-DNA methyltransferase; mFOLFOX, modified fluorouracil, calcium folinate and oxaliplatin; mOS, median overall survival; mpCR, modified pathologic complete response; MPR, major pathologic response; mPFS, median progression-free survival; mRC, metastatic rectal cancer; mRFS, median relapse-free survival; MSI-H, microsatellite instability-high; MSI-L, microsatellite instability-low; MSS, microsatellite stable; MT, mutated; NLM, non-liver metastases; OP, organ preservation; ORR, objective response rate; OS, overall survival; pCR, pathologic complete response; PFS, progression-free survival; pMMR, proficient mismatch repair; RC, rectal cancer; RFA, radiofrequency ablation; RFS, relapse-free survival; SABR, stereotactic ablative radiotherapy; SBRT, stereotactic body radiotherapy; SCRT, short-course radiotherapy; SD, stable disease; WT, wild type; 1L, first-line; 2L, second-line; 3L, third-line.

### Neoadjuvant therapy

The NICHE study first demonstrated impressive outcomes with neoadjuvant nivolumab plus ipilimumab in early-stage colon cancer, reporting a 100% pathologic response rate and 69% pathologic complete response (pCR) rate,^17^ suggesting the potential for complete remission in patients with dMMR/MSI-H locally advanced colon cancer (LACC). These results were confirmed in the NICHE-2 study, where pathologic responses were observed in 99% (106/107) of patients, including a 67% (72/107) pCR rate.^18^ The NICHE-3 trial of nivolumab plus relatlimab reported a pCR rate of 68% and an overall pathologic response rate of 96% in LACC.^19^ The PICC trial of toripalimab plus celecoxib in resectable CRC resulted in a pCR rate of 87%.^20^ Even more notably, atezolizumab plus bevacizumab and short-course radiotherapy (SCRT) achieved a 100% organ preservation (OP) rate in early and intermediate-stage rectal cancer.^21^ Additionally, sintilimab plus IBI310 (anti-cytotoxic T-lymphocyte-associated antigen 4 [anti-CTLA-4]) significantly increased pCR rates compared to sintilimab alone in locally advanced, resectable colorectal cancer (LACRC).^22^

### Adjuvant therapy

In a phase 3 trial involving patients with stage III dMMR colon cancer, the addition of atezolizumab to mFOLFOX6 (modified fluorouracil, calcium folinate, and oxaliplatin) significantly improved DFS compared to chemotherapy alone, establishing this regimen as a new adjuvant standard of care in this setting.^23^

### For pretreated mCRC patients

The CheckMate 142 trial also evaluated nivolumab plus ipilimumab in previously treated mCRC.^24^ Indirect comparisons with nivolumab monotherapy indicated higher response rates and promising 12-month PFS, supporting this combination as a therapeutic alternative. Long-term follow-up confirmed sustained efficacy after 5 years in chemoresistant mCRC.^25^ Not all combination strategies, however, have proven successful. For example, pembrolizumab plus stereotactic ablative radiotherapy (SABR) did not enhance efficacy in programmed cell death protein 1 (PD-1)/programmed death-ligand 1 (PD-L1) blockade-refractory, unresectable mCRC.^26^ To overcome ICI resistance, novel approaches are under investigation, including cadonilimab (anti-PD-1/CTLA-4; NCT05426005) and the combination of botensilimab with balstilimab for refractory mCRC (NCT05608044). Efforts are also underway to expand immunotherapy into earlier-line settings, such as a phase 2 trial of atezolizumab plus IMM-101 (a dendritic cell activator) in oxaliplatin-ineligible stage III CRC patients (NCT05118724).

### Summary for this part

Immunotherapy combinations have transformed the management of dMMR/MSI-H CRC. In the first-line metastatic setting, nivolumab plus ipilimumab has shown superior PFS and HRQoL over chemotherapy, establishing a new SOC. Neoadjuvant dual checkpoint inhibition yields pCR rates of 67%–100% in early-stage disease, suggesting potential for organ preservation in selected patients. For pretreated mCRC, nivolumab plus ipilimumab maintains durable efficacy, though resistance mechanisms necessitate novel agents such as bispecific antibodies. Current research is actively exploring immunotherapy combinations in adjuvant settings and for oxaliplatin-ineligible patients, while also investigating innovative approaches such as combining immunotherapy with radiotherapy or immunomodulators. These developments collectively represent a paradigm shift in dMMR/MSI-H CRC management, where immunotherapy is transitioning from metastatic treatment to potentially curative applications in earlier disease stages. Ongoing clinical trials continue to refine optimal combinations, sequencing, and patient selection criteria to maximize therapeutic benefits.

## Dual immune checkpoint inhibition for pMMR/MSS/MSI-L CRC

### Neoadjuvant therapy

The NICHE study initially reported that nivolumab plus ipilimumab with celecoxib induced pathologic responses in 30% of patients with colon cancer, including a 10% pCR rate.^17^ Alternative dual checkpoint inhibition strategies have shown promise: for example, tremelimumab plus durvalumab demonstrated an acceptable safety profile in patients with mCRC liver metastases.^27^ The NEST-1 trial further supported this approach, suggesting that botensilimab plus balstilimab is an active regimen in resectable CRC.^100^ Based on these encouraging results, the study has been expanded to include two additional cohorts. Recently released data indicate that neoadjuvant botensilimab plus balstilimab is safe—causing no surgical delays—and effective.^28^ Preclinically, high-dose vitamin C (HDVitC) has been shown to enhance cluster of differentiation 8 positive (CD8^+^) T cell infiltration and activation in mouse tumor models, thereby potentiating the efficacy of ICIs.^101^ These mechanistic insights are now being translated into clinical evaluation through the ALFEO trial, which is assessing whether HDVitC can augment the effect of nivolumab plus ipilimumab in colon cancer patients (ECTR2022-502101-15-00).

### For pretreated patients

Botensilimab plus balstilimab has demonstrated promising clinical activity, with an objective response rate (ORR) of 22% and a disease control rate (DCR) of 73%.^29^ Notably, in patients without liver metastases, this combination induced particularly deep and durable responses, including in traditionally difficult-to-treat metastatic sites.^102^ Other dual checkpoint strategies have shown comparable efficacy: pembrolizumab plus muzastotug yielded a superior ORR and median progression-free survival (mPFS) compared with SOC in the third-line setting.^30^ Additionally, the combination of atezolizumab and vilastobart has shown initial evidence of antitumor activity in late-line MSS mCRC.^31^

### Summary for this part

Dual immune checkpoint blockade exhibits modest but promising clinical activity in pMMR/MSS/MSI-L CRC. Although current response rates remain moderate, these results underscore the potential of optimized immunotherapy combinations. Further large-scale trials and biomarker-driven patient selection are warranted to refine these approaches, identify patient subgroups most likely to benefit, and ultimately support regulatory approval for clinical use.

## Combination with chemotherapy regardless of MMR or MSI status

### First-line therapy

The AtezoTRIBE study demonstrated that adding atezolizumab to FOLFOXIRI (fluorouracil, calcium folinate, oxaliplatin, and irinotecan) and bevacizumab significantly improved PFS in mCRC.^32^ A post-hoc analysis indicated that this PFS benefit was statistically significant in the dMMR subgroup but not in pMMR patients. In contrast, the CheckMate 9X8 trial, which evaluated nivolumab plus FOLFOX and bevacizumab, did not show significant PFS improvement over standard therapy.^33^ Nevertheless, both studies consistently reported enhanced DCR in the pMMR subgroup. Additionally, the NIVACOR study provided promising preliminary data, showing that nivolumab combined with FOLFOXIRI and bevacizumab exhibited clinical activity in advanced mCRC patients with rat sarcoma viral oncogene homolog (RAS) or B-Raf proto-oncogene, serine/threonine kinase (BRAF) mutations, with particularly encouraging responses observed in the pMMR population.^34^

### Neoadjuvant therapy

In the OPTICAL-2 trial, cadonilimab plus mFOLFOXIRI (modified fluorouracil, calcium folinate, oxaliplatin, and irinotecan) resulted in higher pCR, downstaging, and major pathological response (MPR) rates compared with FOLFOX chemotherapy in patients with LACRC.^35^ These findings suggest that combining intensified chemotherapy with dual immunotherapy may represent a promising strategy for improving outcomes in this setting.

### Second-line or later-line therapy

Camrelizumab combined with irinotecan, surufatinib, and granulocyte-macrophage colony-stimulating factor (GM-CSF) demonstrated notable antitumor activity as a second-line regimen in advanced CRC.^36^ Furthermore, in patients with colorectal liver metastases (CRLMs), adjuvant sintilimab plus hepatic arterial infusion chemotherapy (HAIC) and regorafenib showed promising efficacy, achieving a 1-year relapse-free survival (RFS) rate of 67.8% in high-risk patients.^37^ Building on these results, ongoing research is evaluating novel neoadjuvant strategies, including a phase 2 trial of pucotenlimab (anti-PD-1) combined with CAPEOX and bevacizumab in high-risk resectable MSS CRLM (ChiCTR2400085958).

### Summary

In dMMR/MSI-H mCRC, combining immunotherapy with chemotherapy represents a valid first-line option, supported by significant PFS benefits. For pMMR/MSS mCRC, although PFS improvements remain limited, enhanced disease control and stabilization suggest a potential role for these combinations in delaying progression, particularly in patients with RAS/BRAF mutations or liver-limited disease. Later-line strategies that incorporate anti-angiogenic agents may provide additional benefit by modulating the TME. Future efforts should focus on biomarker-guided patient selection and the development of novel combination regimens to improve outcomes in pMMR/MSS CRC.

## Combination with chemotherapy for pMMR/MSS/MSI-L CRC

### First-line therapy

In patients with RAS-mutant unresectable mCRC, the combination of tremelimumab, durvalumab, and mFOLFOX6 has shown robust clinical activity, achieving a 3-month PFS rate of 90.7% and an ORR of 64.5%.^38^

### Neoadjuvant therapy

Tislelizumab plus CAPEOX has demonstrated notable efficacy in high-risk LACC, increasing the pCR rate to 51.85%.^39^ Encouraging results have also been observed in LARC, where cadonilimab combined with CAPEOX yielded a pCR rate of 15.4% and a major pathological response (MPR) rate of 46.2%, supporting its potential as an organ-preserving strategy.^40^ In another LARC study, anti-PD-1 antibody combined with rectal arterial infusion of oxaliplatin following induction chemotherapy resulted in a pCR rate of 35.5% and an MPR rate of 77.4%.^41^ Additionally, a trial evaluating dostarlimab plus CAPEOX in T4N0 or stage III resectable colon cancer is currently recruiting (NCT06567782).

### For pretreated patients

Early trials of immunotherapy combinations in previously treated mCRC yielded largely negative results. A phase 2 study of nivolumab plus trifluridine-tipiracil (FTD/TPI, TAS-102) reported no objective responses and was terminated early.^42^ Similarly, pembrolizumab combined with azacytidine showed minimal activity (ORR: 3%) in chemotherapy-refractory mCRC, leading to premature study closure.^43^ More promising outcomes have been observed with biomarker-selected approaches. The MAYA trial demonstrated that temozolomide (TMZ) priming followed by low-dose ipilimumab and nivolumab can induce durable clinical benefit in patients with O6-methylguanine-DNA methyltransferase (MGMT)-silenced mCRC.^44^ This strategy was further validated in the ARETHUSA trial, where four of six MGMT-silenced, RAS-mutant mCRC patients achieved stable disease following TMZ priming and pembrolizumab.^103^ Additionally, sintilimab combined with chidamide and bevacizumab exhibited encouraging efficacy in heavily pretreated mCRC patients who had failed ≥2 prior lines of therapy.^45^

### Summary

First-line immunotherapy combined with chemotherapy shows considerable potential in RAS-mutant mCRC and warrants validation in phase 3 trials. In the neoadjuvant setting, such combinations may expand organ preservation options in LARC and LACC, with pCR rates comparable to traditional regimens. Success in later-line treatment depends on precision strategies, including targeting MGMT silencing or histone deacetylase (HDAC) inhibition. Further development of predictive biomarkers and optimization of treatment sequencing will be essential to maximize clinical benefit in pMMR/MSS/MSI-L CRC.

## The impact of chemotherapy on the TME

### Immunogenic effects of conventional chemotherapeutics

**Figure 1:**
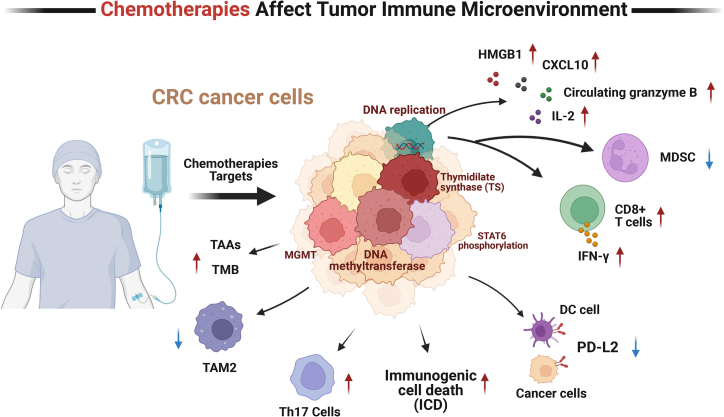
Mechanisms of chemotherapy in modulating the tumor immune microenvironment of colorectal cancer Schematic representation of how conventional chemotherapeutic agents influence the tumor immune microenvironment in colorectal cancer (CRC). Chemotherapies such as 5-fluorouracil, oxaliplatin, and irinotecan act on multiple cellular and molecular targets—including cancer cells, myeloid-derived suppressor cells (MDSCs), dendritic cells (DCs), and CD8^+^ T cells—leading to immunogenic cell death, enhanced T cell infiltration and activation, altered cytokine/chemokine signaling, and modulation of immune checkpoint expression. Key immunomodulatory outcomes include upregulation of IFN-γ, granzyme B, and CXCL10, as well as changes in PD-L2 expression and MDSCs frequency, collectively contributing to an anti-tumor immune response. TAM2, type-2 tumor-associated macrophages; MGMT, O^6^-methylguanine-DNA methyltransferase. ↑ up-regulated, ↓ down-regulated.

5-Fluorouracil (5-FU) exerts multiple immunomodulatory effects, including reducing myeloid-derived suppressor cells (MDSCs), enhancing CD8^+^ T cell infiltration, and boosting interferon-γ (IFN-γ)-dependent antitumor responses (Figure 1).^104^ It also upregulates PD-L1 expression on tumor cells, which may sensitize tumors to ICIs.^105^ Oxaliplatin induces immunogenic cell death (ICD), characterized by calreticulin exposure and high-mobility group box 1 (HMGB1) release.^106^ Additionally, it promotes dendritic cell (DC) maturation, downregulates programmed death-ligand 2 (PD-L2) expression, reduces MDSCs, and increases the CD8^+^/regulatory T cells (Tregs) ratio.^107^

### Combination strategies and synergistic effects

Although irinotecan primarily acts through topoisomerase I inhibition to induce cell death, both 5-FU and irinotecan can upregulate epidermal growth factor receptor (EGFR) expression and enhance cetuximab-mediated antibody-dependent cellular cytotoxicity (ADCC), independent of kirsten rat sarcoma viral oncogene homolog (KRAS) status.^108^ The FOLFOX regimen reduces Tregs and MDSCs and, when combined with bevacizumab, increases T helper 17 (Th17) cell frequency.^109^ Furthermore, FOLFOX plus pembrolizumab elevates C-X-C motif chemokine 10 (CXCL10) levels and granzyme B production, indicating enhanced T cell activity.^110^ Similarly, FOLFIRI (fluorouracil, calcium folinate, and irinotecan) combined with cetuximab promotes DC activation, improves antigen presentation, and elicits robust cytotoxic T lymphocyte (CTL) responses.^111^

### Novel chemotherapeutic agents and epigenetic modulators

FTD/TPI induces ICD in MSS CRC. In combination with oxaliplatin, it reduces type-2-tumor-associated macrophage (TAM2) polarization while enhancing CD8^+^ T cell infiltration and activation.^112^ Epigenetic modulators such as azacitidine and decitabine reactivate silenced tumor-associated antigens (TAAs) and, when combined with ICIs, reduce MDSCs while increasing CD8^+^ T cell infiltration.^113^ Temozolomide (TMZ) induces hypermutation in MGMT-silenced tumors, generating dMMR/MSI-H-like phenotypes that become susceptible to ICIs.^44^^,^^103^ Chidamide, a histone deacetylase inhibitor, enhances natural killer (NK) and CTL activity, upregulates PD-L1 and major histocompatibility complex (MHC) expression, and reduces MDSCs.^114^ Collectively, these findings indicate that conventional cytotoxic agents—including 5-FU, irinotecan, oxaliplatin, and TMZ—can promote tumor hypermutation and immunogenicity, providing a strong rationale for developing innovative chemo-immunotherapy strategies in CRC.

## Combination with targeted therapy for pMMR/MSS/MSI-L CRC

### Anti-epidermal growth factor receptor

Combining anti-EGFR antibodies with immunotherapy has shown promise in pretreated RAS/BRAF wild-type (RAS/BRAF^WT^) pMMR/MSS mCRC. In the CAVE trial, cetuximab rechallenge plus avelumab was evaluated in chemotherapy-refractory RAS wild-type (RAS^WT^) mCRC, of which 92% (71/77) had MSS tumors.^46^ The results supported this combination as an effective rechallenge strategy, with subsequent analyses linking skin toxicity and a baseline neutrophil-to-lymphocyte ratio (NLR) < 3 to improved survival outcomes.^115^^,^^116^ Similarly, the LCCC1632 trial investigated nivolumab and ipilimumab plus panitumumab in pretreated KRAS/NRAS/BRAF^WT^ mCRC, reporting a 12-week response rate of 35%, which met the prespecified primary endpoint and supports further investigation.^47^

## Anti-vascular endothelial growth factor/vascular endothelial growth factor receptor

### First-line therapy

Sintilimab plus anlotinib showed encouraging antitumor activity in advanced mCRC, with an ORR of 48.3%.^48^

### For pretreated patients

**(1) Second-line or later:** the LEAP-017 study reported that pembrolizumab plus lenvatinib improved PFS and ORR versus standard of care, with a trend toward OS benefit, including in Asian patients.^49^ Nivolumab plus ipilimumab and regorafenib (REG) demonstrated durable clinical benefit, particularly in chemotherapy-resistant patients without liver metastases.^50^ The phase 3 STELLAR-303 trial is currently evaluating atezolizumab plus XL092 in mCRC (NCT05425940). **(2) Third-line or later:** the CAMILLA trial showed encouraging efficacy with durvalumab plus cabozantinib in chemotherapy-refractory advanced CRC.^51^ The REGONIVO regimen (nivolumab plus regorafenib) also exhibited significant antitumor activity.^52^ The LEAP-005 study further validated pembrolizumab plus lenvatinib in this setting.^53^

### Summary for anti-VEGF/VEGFR

Immunotherapy combined with anti-angiogenic agents is primarily used in pretreated pMMR/MSS mCRC. Although first-line evidence remains limited, sintilimab plus anlotinib suggests the potential of PD-1/ vascular endothelial growth factor receptor (VEGFR) dual blockade in untreated advanced disease. Dual PD-1/VEGF inhibition represents a viable strategy, particularly in RAS/BRAFWT or non-liver metastatic subgroups. Triple combinations (e.g., PD-1/VEGF/mitogen-activated protein kinase kinase (MEK) inhibition) may further enhance efficacy but require biomarker-guided selection. More first-line trials are warranted, alongside future efforts to validate predictive biomarkers, profile metastatic sites, and explore novel anti-angiogenic targets to remodel the immunosuppressive TME.

## Mitogen-activated protein kinase kinase inhibition

Spartalizumab combined with trametinib (an MEK inhibitor) and dabrafenib (a BRAF inhibitor) achieved a 25% response rate in BRAF^V600E^ MSS mCRC across first-line or later treatment settings.^54^ In contrast, durvalumab plus trametinib as second-line or later therapy did not meet predefined efficacy criteria (ORR: 3.4%).^55^ Notably, among three patients with concurrent lung and liver metastases, clinical benefit was confined to lung lesions, suggesting that metastatic site location critically influences treatment outcomes. This may be attributable to liver-macrophage-mediated T cell depletion, prompting proposals to combine liver-directed radiotherapy with immunotherapy to overcome this limitation.^117^ The IMblaze370 trial also showed that atezolizumab plus cobimetinib did not improve OS versus regorafenib or atezolizumab monotherapy in the third-line setting,^56^ underscoring the need for biomarker-driven patient selection.

### Summary for MEK inhibition

While immunotherapy plus MEK inhibition shows limited efficacy in unselected pMMR/MSS CRC, activity persists in BRAF-mutant and non-liver metastatic subgroups. Future studies should develop biomarkers based on mitogen-activated protein kinase (MAPK) pathway activation, address liver-specific immunosuppression (e.g., via liver-directed radiotherapy), and explore rational combinations co-targeting complementary pathways such as EGFR or human epidermal growth factor receptor 2 (HER2).

## The impact of targeted therapy on the TME

### Anti-EGFR therapies

**Figure 2:**
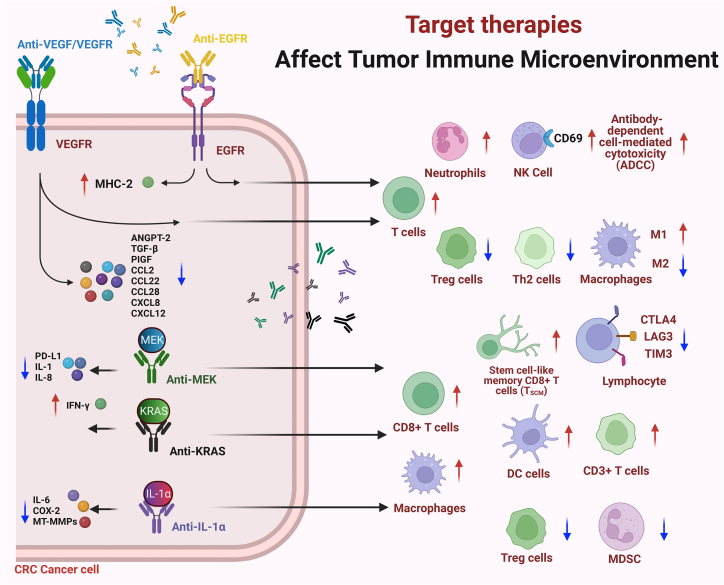
Mechanisms of targeted therapies in remodeling the tumor immune microenvironment of colorectal cancer Schematic overview of how targeted therapies modulate the tumor immune microenvironment in colorectal cancer. Key therapeutic classes—including anti-VEGF/VEGFR, anti-EGFR, and MEK inhibitors—act on specific molecular targets to alter immune cell composition and function. These interventions promote CD8^+^ T cell and NK cell activity, enhance antigen presentation via MHC class II, suppress immunosuppressive cells, and modulate chemokine/cytokine networks. Combinatorial effects also include the induction of stem-like memory CD8^+^ T cells (T_SCM_) and altered expression of immune checkpoints such as LAG-3, TIM-3, and CTLA-4, collectively fostering an immunostimulatory tumor milieu. ↑ up-regulated, ↓ down-regulated.

Cetuximab modulates the immune response primarily by triggering NK-cell-mediated ADCC and facilitating DC-NK cell crosstalk, which enhances MHC class II expression and promotes T cell infiltration (Figure 2).^46^ Its synergy with avelumab is attributed to their shared immunoglobulin G1 (IgG1) isotype and capacity to induce ADCC.^46^ In EGFRvIII-mutated CRC—a tumor-specific variant—extracellular adenosine triphosphate (ATP) is preferentially metabolized into immunosuppressive adenosine; this resistance mechanism may be counteracted by adenosine pathway blockade.^118^ In contrast, panitumumab exhibits weaker ADCC activity but can stimulate neutrophil- and monocyte-mediated immune responses.^119^ Both cetuximab and panitumumab also display anti-angiogenic properties.^120^

### Anti-angiogenic agents

Beyond targeting tumor vasculature directly, anti-angiogenic agents remodel the TME by promoting vascular normalization, which improves T cell infiltration and alleviates hypoxia.^121^ These therapies also reverse VEGF-induced suppression of DC maturation and prevent CD8^+^ T cell exhaustion.^122^^,^^123^ Furthermore, they disrupt immunosuppressive circuits, particularly the TAM2/Th2/Tregs-ANGPT2 angiogenesis axis.^124^ Interestingly, lower doses of anti-angiogenic drugs may be more effective at inducing vascular normalization, polarizing macrophages toward an M1 phenotype, and enhancing cluster of differentiation 4 positive (CD4^+^)/CD8^+^ T cell infiltration.^125^ However, certain multi-kinase inhibitors such as sorafenib can partially suppress DC function, potentially compromising their combinatory utility with immunotherapy.^126^

### MEK inhibition

MEK inhibitors reshape the TME by increasing infiltration of effector CD8^+^ T cells while reducing T cell exhaustion and promoting their activation.^127^ Cobimetinib, for example, upregulates MHC class I expression, recruits CD8^+^ T cells, and suppresses immunosuppressive cytokine networks.^56^^,^^127^ Nevertheless, combining atezolizumab with cobimetinib failed to show superior efficacy, potentially due to compensatory MAPK pathway activation.^56^ Notably, MEK inhibition promotes the formation of stem-cell-like memory CD8^+^ T (T_SCM_) cells, which may contribute to sustained antitumor immunity.^128^

## Combination with chemotherapy and targeted therapy for pMMR/MSS/MSI-L CRC

### First-line therapy

In advanced RAS/BRAF^WT^ mCRC, sintilimab combined with chemotherapy and cetuximab achieved high ORR (86.7%) and DCR (100%).^57^ The BBCAPX trial further validated sintilimab plus CAPEOX and bevacizumab in RAS-mutant mCRC, demonstrating an ORR of 84%, DCR of 100%, and mPFS of 17.9 months.^58^ Notably, patients with liver metastases showed better outcomes than those with metastases at other sites. Based on these results, a phase 3 trial has been initiated to further evaluate this regimen’s efficacy, safety, and predictive biomarkers (NCT05171660).

Pembrolizumab combined with CAPEOX and bevacizumab have consistently demonstrated antitumor activity in both RAS-mutated mCRC and tumors with high immune infiltration.^59^ Similarly, serplulimab plus CAPEOX and HLX04 (a bevacizumab biosimilar) resulted in longer PFS than placebo plus CAPEOX and bevacizumab (17.2 vs. 10.7 months) in mCRC.^60^

### Neoadjuvant therapy

In MSS LARC, camrelizumab plus mFOLFOXIRI and bevacizumab achieved a CR rate of 44.8%.^61^ The AMBITION trial is evaluating camrelizumab plus mFOLFOX6 and apatinib in right-sided LACC (NCT04625803), and the BASKETⅢ study is assessing sintilimab plus mFOLFOX6 and bevacizumab in LACRC (NCT06791512), reflecting an important expansion of immunotherapy into earlier-stage disease.

### For pretreated patients

**(1) Second-line or later:** toripalimab combined with oxaliplatin/irinotecan and surufatinib showed encouraging efficacy in RAS/BRAF-mutant mCRC.^62^ Comparable outcomes were observed with penpulimab plus irinotecan and anlotinib.^63^ **(2) Third-line or later:** envafolimab plus irinotecan and cetuximab yielded notable efficacy in RAS/BRAF^WT^ mCRC.^64^ Tislelizumab with the same backbone achieved an ORR of 33% in RAS^WT^ disease.^65^ Additionally, enlonstobart (anti-PD-1) combined with irinotecan and becotatug (anti-EGFR) induced promising responses in RAS/BRAF^WT^ non-dMMR/MSI-H mCRC.^66^ **(3) Limitations and negative findings:** the AVETUXIRI trial of avelumab plus irinotecan and cetuximab in chemotherapy-refractory (and anti-EGFR refractory if RAS^WT^) mCRC did not meet its efficacy endpoint.^67^ Similarly, the BACCI trial showed limited benefit from adding atezolizumab to capecitabine and bevacizumab in refractory mCRC, though some activity was observed in patients without liver metastases.^68^

### Summary

The integration of immunotherapy with chemotherapy and targeted therapy represents a new paradigm for treating pMMR/MSS CRC. RAS/BRAF^WT^ patients derive substantial benefit from immunotherapy combined with chemotherapy and anti-EGFR therapy, whereas RAS/BRAF-mutant patients and those with liver metastases respond better to regimens incorporating anti-angiogenic agents—potentially due to angiogenesis inhibition remodeling the TME. Nevertheless, treating refractory disease remains challenging, underscoring the need for further biomarker development and optimized combination strategies.

## Combination with radiotherapy for pMMR/MSS/MSI-L CRC

Initial clinical investigations from a phase 1b trial indicated that multisite stereotactic body radiotherapy (SBRT) followed by pembrolizumab was well tolerated in patients with pMMR mCRC.^69^ However, a subsequent phase 2 trial showed that combining atezolizumab with SBRT did not improve PFS in heavily pretreated mCRC, regardless of MMR status.^70^ More encouraging results came from another phase 2 trial, in which radiotherapy combined with ipilimumab and nivolumab as third-line or later therapy demonstrated both feasibility and durable clinical activity, providing important proof of concept for this approach.^71^ In contrast, other combinations have shown limited efficacy. The addition of durvalumab and tremelimumab to concurrent radiotherapy in chemotherapy-refractory mCRC did not meet predefined efficacy thresholds for further development.^72^ Together, these findings highlight the need for novel strategies to enhance the efficacy of radio-immunotherapy in mCRC.

### The impact of radiotherapy on the TME

**Figure 3:**
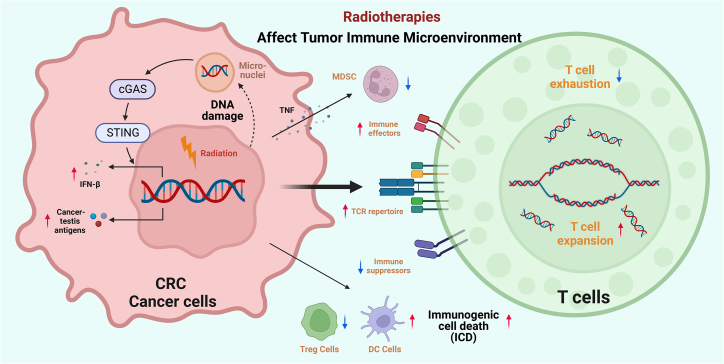
Mechanisms of radiotherapy in remodeling the tumor immune microenvironment of colorectal cancer Schematic representation of radiotherapy-induced immunomodulation in colorectal cancer. Radiation triggers DNA damage and immunogenic cell death, activating the cGAS-STING pathway and enhancing type I interferon signaling. These events promote dendritic cell maturation and antigen presentation, expand the T cell receptor (TCR) repertoire, and reduce immunosuppressive cell populations such as Tregs and MDSCs. Concurrently, radiotherapy alleviates T cell exhaustion and augments the infiltration and function of immune effector cells, collectively converting the tumor microenvironment toward an immunostimulatory state. ↑ up-regulated, ↓ down-regulated.

Radiation-induced cytosolic DNA acts as a potent immune stimulus by activating the cyclic GMP-AMP synthase (cGAS)-stimulator of interferon genes (STING) pathway, which initiates downstream immune signaling and robustly enhances interferon-β (IFN-β) secretion (Figure 3).^129^ Local high-dose radiotherapy amplifies this antitumor response by increasing type I interferon production, thereby improving tumor-infiltrating dendritic cell (TIDC) cross-priming and CD8^+^ T cell activation.^130^ Beyond localized effects, radiotherapy also exerts systemic immunomodulation: it promotes effector T cell infiltration into tumors and enhances CD8^+^ T cell priming in draining lymphoid tissues, strengthening systemic antitumor immunity.^131^ Moreover, both radiotherapy alone and radio-chemotherapy can induce ICD, fostering a pro-immunogenic TME.^132^ The immunostimulatory potential of radiotherapy is further enhanced when combined with ICIs. This strategy expands oligoclonal T cell populations, reduces immunosuppressive Tregs and MDSCs, reverses T cell exhaustion, and significantly improves cross-priming of tumor-specific CTLs.^133^ Collectively, these effects establish an immunologically favorable milieu that supports more effective disease control.

## Combination with radiotherapy and chemotherapy for pMMR/MSS/MSI-L LARC neoadjuvant therapy

Chemoradiotherapy (CRT) combined with nivolumab resulted in a modified pCR (mpCR) rate of 65%.^73^ In the POLARSTAR trial, post-hoc analysis indicated that tislelizumab plus CRT significantly improved pathological tumor regression and facilitated successful total mesorectal excision.^74^ Additionally, tislelizumab added to long-course CRT increased the pCR rate to 46.67% in high-risk, mesorectal fascia positive (MRF^+^) locally advanced low rectal cancer (LALRC).^134^

The TORCH trial demonstrated that toripalimab combined with CAPEOX and short-course radiotherapy (SCRT) achieved a CR rate of 55.8%, supporting its potential as an organ-preserving strategy.^75^ Emerging radiotherapy approaches also show promise. Split-course hypofractionated radiotherapy (HFRT) combined with CAPEOX and tislelizumab led to a pCR rate of 56.5%.^76^ HFRT combined with chemotherapy and toripalimab also markedly improved objective response rates (82.9% and 65.4%) in pMMR/MSS locally recurrent rectal cancer (LRRC) compared with historical benchmarks.^77^ Lastly, serplulimab plus FOLFOXIRI and long-course radiotherapy (LCRT) significantly increased CR rates (75%) and anal preservation rates (95%) in LALRC.^78^ Notably, SCRT-based immunochemotherapy regimens appear to yield higher pCR/CR rates than LCRT-based combinations in pMMR/MSS rectal adenocarcinoma.^135^ To further optimize efficacy while preserving T cell function, ongoing trials are evaluating node-sparing SCRT combined with CAPEOX and tislelizumab (NCT05972655 and NCT06507371), representing an important direction in neoadjuvant therapy.

### Summary

The integration of immunotherapy with radiotherapy and chemotherapy represents a transformative approach in the neoadjuvant treatment of pMMR/MSS LARC, enabling notable pCR rates and enhancing organ preservation. Future efforts should focus on refining radiation techniques, identifying predictive biomarkers, and validating these strategies in larger randomized trials to broaden clinical applicability.

## Combination with radiotherapy and targeted therapy for pMMR/MSS/MSI-L CRC

### Neoadjuvant therapy for LARC

Toripalimab combined with fruquintinib and SCRT achieved a pCR rate of 37.5%.^79^ Similarly, atezolizumab plus bevacizumab and SCRT resulted in an OP rate of 42% in patients with early or intermediate-stage rectal cancer.^21^

### For pretreated patients

In later-line settings, the RIFLE trial reported that tislelizumab combined with fruquintinib and SABR yielded an ORR of 34.3% in mCRC.^80^ The FRUIT trial also demonstrated activity for a similar regimen (tislelizumab/fruquintinib/SBRT) in third-line or later mCRC, with an ORR of 26%.^81^ A retrospective analysis further indicated that combining immunotherapy and anti-angiogenic agents with radiotherapy significantly improved survival outcomes when used as posterior-line therapy for pMMR/MSS mCRC, underscoring the potential of this multimodal approach even in refractory disease.^136^

### Summary

The combination of immunotherapy, radiotherapy, and targeted therapy (primarily anti-angiogenic agents) represents a promising strategy for pMMR/MSS/MSI-L CRC, with encouraging responses in both locally advanced and metastatic settings. Future trials should focus on identifying predictive biomarkers and optimizing radiation modalities to broaden clinical applicability and overcome resistance.

## Combination with chemotherapy, targeted therapy, and radiotherapy for pMMR/MSS/MSI-L CRC

In the UNION mRC study, SCRT followed by tislelizumab and CAPEOX, combined with either bevacizumab or cetuximab, demonstrated encouraging efficacy and a manageable safety profile as first-line therapy for unresectable pMMR/MSS metastatic rectal cancer.^82^ The UNION TNT trial evaluated SCRT followed by adebrelimab and CAPEOX plus fruquintinib as a total neoadjuvant therapy in high-risk LARC, reporting promising CR (65%) and pCR (63%) rates, with an overall manageable safety profile.^83^ In the ZZU-1 study, neoadjuvant treatment with SCRT followed by sintilimab, CAPEOX, and anlotinib achieved a CR rate approaching 100% in pMMR/MSS LARC patients, surpassing outcomes reported in previous studies.^84^ Close monitoring for adverse events remains necessary throughout treatment.

## Promising candidates for combination immunotherapies

### New immune checkpoint inhibitors

Early-phase trials of next-generation ICIs in advanced CRC have shown limited but variable activity. In a phase 1/1b trial, sabatolimab (anti-T cell immunoglobulin and mucin domain-containing protein 3, anti-TIM-3) combined with spartalizumab elicited partial responses in two patients with advanced CRC.^85^ Additionally, the co-formulated favezelimab (anti-lymphocyte activation gene 3 protein, anti-LAG-3)/pembrolizumab did not improve OS compared with SOC in previously treated PD-L1-positive (combined positive score [CPS] ≥1) MSS/pMMR mCRC, although the combination of favezelimab and pembrolizumab has shown promising antitumor activity in earlier assessments.^86^ Several ongoing trials are evaluating novel ICIs in CRC and other solid tumors, including anti-LAG3 antibody (NCT03642067, NCT03867799, and NCT05328908) and anti-T cell immune receptor with immunoglobulin and ITIM domains (anti-TIGIT) antibody (NCT04929223 and NCT04895722).

### Bispecific antibody

Ivonescimab (a PD-1/VEGF bispecific antibody) combined with ligufalimab (anti-CD47) and FOLFOXIRI has shown promising efficacy as first-line treatment in MSS mCRC patients.^87^ In the second-line or later setting, IBI363 (a PD-1/interleukin-2 [IL-2] bispecific antibody) plus bevacizumab exhibited encouraging clinical activity in advanced non-MSI-H/dMMR CRC.^88^ Similarly, amivantamab (targeting EGFR and mesenchymal epithelial transition [MET] receptors) demonstrated antitumor activity in third- or fourth-line treatment.^137^

### KRAS, BRAF, and poly-ADP-ribose polymerase inhibitors

KRAS^G12C^ inhibitors—including sotorasib, adagrasib, olomorasib, and MK-1084—have demonstrated meaningful antitumor activity in previously treated patients with KRAS^G12C^-mutant mCRC, representing a significant advance for this historically difficult-to-treat population.^138^ In the first-line setting, the ongoing SEAMARK trial is evaluating pembrolizumab combined with encorafenib (a BRAF^V600E^ inhibitor) and cetuximab in treatment-naïve patients with BRAF^V600E^-mutant dMMR/MSI-H CRC (NCT05217446). Preliminary data also suggest that nivolumab plus encorafenib and cetuximab exhibits promising efficacy in refractory BRAF^V600E^ MSS mCRC,^89^ prompting initiation of a phase 2 trial of this triple-combination regimen in a similar patient group (NCT05308446). The therapeutic landscape is further expanding to include DNA damage response pathways. Ongoing trials are evaluating pembrolizumab plus olaparib (a PARP inhibitor) in homologous recombination repair deficiency (HRD)-positive mCRC (NCT05201612), as well as durvalumab combined with olaparib and cediranib (a VEGFR inhibitor; NCT02484404). However, the DAPPER trial reported limited antitumor activity with durvalumab plus either olaparib or cediranib in advanced pMMR CRC,^90^ underscoring the need for biomarker-guided patient selection in such combination strategies.

### Oncolytic virus

Preliminary results from the GOBLET study indicate that pelareorep, in combination with atezolizumab and chemotherapy, may elicit protective immune responses in patients with MSS mCRC receiving third-line treatment.^91^ In a phase 1/2 trial, PexaVec combined with durvalumab and tremelimumab also showed potential efficacy in refractory pMMR mCRC.^92^ Further advancing the field, an ongoing phase 2 trial is evaluating RP2/RP3 oncolytic herpes simplex viruses in combination with atezolizumab and bevacizumab for previously treated pMMR/MSS CRC (NCT05733611).

### Vaccine

Vaccine-based immunotherapy has shown initial clinical promise in CRC. In the NOUS-209-01 study, pembrolizumab combined with the Nous-209 vaccine as first-line treatment elicited objective responses in five adults with dMMR/MSI-H locally advanced or metastatic CRC, providing proof of concept for vaccine strategies in immunogenic subtypes.^93^ In the adjuvant setting, a first-in-human trial of the ELI-002 2P vaccine in high-risk KRAS^G12D/G12R^-mutated CRC confirmed its safety and immunogenicity, with clear vaccine-induced immune responses observed.^139^ Encouraging activity has also been reported in traditionally immunotherapy-resistant MSS tumors. Early studies indicated that nivolumab plus ipilimumab combined with a vaccine improved OS in selected pretreated MSS patients, and updated data suggest potential benefit even in first-line MSS mCRC.^94^ However, not all combinations have succeeded: atezolizumab plus polyPEPI1018 did not induce objective tumor responses in relapsed/refractory MSS mCRC.^95^ Ongoing studies continue to evaluate novel vaccine platforms, including AlloStim with anti-PD-L1 as fourth-line therapy for pMMR/MSS mCRC (NCT06557278), and a mutant KRAS vaccine combined with balstilimab and botensilimab in the maintenance setting (NCT06411691).

### Adoptive cell therapy

A phase 3 trial has demonstrated that adoptive cell therapy (ACT) combined with CAPEOX and bevacizumab as first-line treatment significantly improves both PFS and OS in mCRC, establishing ACT as a potentially transformative option in frontline management.^96^ In the chimeric antigen receptor (CAR) T cell arena, anti-CEA CAR-T therapy prolonged RFS in postoperative CRLM patients,^140^ while GCC19CART demonstrated significant clinical activity and durable responses in refractory mCRC.^141^ Additionally, patient enrollment has been completed for a trial evaluating WU-NK-101 combined with cetuximab as second-line or later therapy for advanced/metastatic CRC (NCT05674526). The forthcoming results are anticipated to further clarify the utility of NK-cell-based therapies in treatment-resistant disease.

### Targeting tumor-associated macrophages

The anti-IL-1α antibody MABp1 represents a novel strategy for reprogramming the immunosuppressive TME by selectively blocking pro-tumor functions of tumor-associated macrophages (TAMs). Clinical studies indicate its potential utility in refractory advanced CRC, offering a mechanism-based approach to modulate the TME.^142^ In parallel, a phase 2 trial (daNIS-3) evaluating tislelizumab plus FOLFIRI/mFOLFOX and NIS793 (an anti-TGF-β antibody) as second-line therapy for MSS mCRC had been initiated (NCT04952753).^143^ However, the study was terminated prematurely due to safety concerns.

### Other approaches

The CAIRE study reported that durvalumab plus tazemetostat (an enhancer of zeste homolog 2 (EZH2) inhibitor) achieved a DCR of 35.3% in advanced MSS CRC after at least one prior therapy, suggesting epigenetic modulation may help overcome immunotherapy resistance.^97^ In chemotherapy-resistant pMMR/MSS mCRC, balstilimab (anti-PD-1) plus CR6068 (an prostaglandin E2 receptor 4 [EP4] antagonist) induced durable responses.^98^ The phase 2 RENMIN-215 trial introduced an innovative strategy combining fecal microbiota transplantation (FMT) with tislelizumab and fruquintinib as third-line or later treatment for MSS mCRC, showing improved survival outcomes and supporting a potential role for gut microbiome modulation in overcoming immunotherapy resistance.^99^ Early-phase studies have also explored innate immune activation: a phase 1 trial of dazostinag (an STING agonist) plus pembrolizumab demonstrated promising antitumor activity, leading to the initiation of an expanded CRC cohort,^144^ which may offer new avenues for immune-refractory MSS disease.

## Biomarkers or clinical parameters to predict immunotherapy response in CRC

### For dMMR/MSI-H CRC

High MSI and elevated tumor mutational burden (TMB) are well-established predictors of improved response to immunotherapy in dMMR/MSI-H CRC.^145^ However, TMB and MSIsensor/MSICare scores alone may not reliably predict efficacy.^146^ Increased expression of human leukocyte antigen (HLA) genes, particularly components of the MHC class II complex, is also associated with improved outcomes.^147^ Other promising biomarkers include T cell/B cell receptor diversity and the neoantigen presentation score (NEOPS), both linked to immunotherapy benefit,^148^ whereas high neutrophil-to-lymphocyte ratio (NLR) and elevated pan-immune-inflammation value (PIV) suggest primary resistance.^148^^,^^149^

Right-sided primary tumors generally respond better to immunotherapy, while elevated CEA levels predict poorer outcomes.^150^ Circulating tumor DNA (ctDNA) dynamics serve as an effective real-time marker of treatment response.^151^ A prognostic model integrating quantitative imaging features from baseline computed tomography (CT) scans and clinical factors may help identify patients most likely to benefit from combined anti-PD-1/anti-CTLA-4 therapy.^152^

A simplified classifier incorporating 19 microsatellite markers and TGF-β-related RNA markers shows utility in predicting immune response evaluation criteria in solid tumors (RECIST) PFS (iPFS).^146^ By contrast, CMSs classification has limited predictive value in this context, likely due to tumor heterogeneity.^153^ KRAS, BRAF, and beta-2-microglobulin (B2M) mutations are not associated with shorter iPFS, nor are transcriptomic signatures reflecting TME composition, single-gene expression, or pathway activity—including gene sets related to angiogenesis, epithelial-mesenchymal transition (EMT), TGF-β signaling, tumor necrosis factor (TNF), interferon, or mammalian target of rapamycin (mTOR).

### For pMMR/MSS/MSI-L CRC

POLE/POLD1 mutation with ultra-hypermutated phenotype, particularly loss-of-proofreading (LOP) variants, enhance ICI sensitivity.^154^ Immunoscore IC represents a promising tool for patient selection.^32^ Although conventional TMB has limited predictive value in this subgroup, modified TMB (mTMB)^155^ and plasma TMB (≥28 mut/Mb)^156^ show improved performance. Additional predictive factors include HLA supertypes B8 and A3, which identify patients more likely to benefit from adding ICIs to FOLFOXIRI/bevacizumab^157^; EGFRvIII mutation, which guides cetuximab-immunotherapy combinations^118^; the VHIO immune gene-expression signature (VIGex) for identifying potential responders^158^; C-reactive protein (CRP), especially when early oxaliplatin-based chemotherapy mitigates tumor-associated inflammation^159^; and magnetic resonance imaging (MRI)-based radiomic features for predicting pCR in LARC receiving neoadjuvant immunotherapy with CRT.^160^

Integrating adverse-events-derived biomarkers with molecular markers improves predictive accuracy in refractory pMMR CRC.^161^ An immune-activation-related risk score based on six immune-related genes effectively stratifies pMMR/MSS patients with dMMR-like immune features and predicts immunotherapy adaptability.^162^ A subset of MSS/TMB-low CRC patients with favorable antitumor immunity and a distinct mutational profile may also derive benefit from immunotherapy.^163^

### Metastatic site considerations

Metastatic location influences immunotherapy outcomes in both dMMR and pMMR tumors. Immunoediting remodels the metastatic genome during therapy, and immunoediting scores show predictive potential.^164^ Liver metastases in MSS CRC are enriched for neutrophils, which may contribute to reduced ICI efficacy.^165^ In dMMR/MSI-H and/or TMB-high mCRC, peritoneal metastases with malignant ascites correlate with poorer OS,^166^ whereas immunotherapy combined with primary tumor resection yields the best OS in MSI-H CRC with isolated peritoneal disease.^167^ In MSS CRC with abdominal metastases, TMB >8.0 or BRAFV^600E^ mutation is associated with prolonged PFS in patients receiving first-line alternating oxaliplatin-based chemotherapy and nivolumab.^168^ This regimen is particularly effective for unresectable liver or lymph node metastases from right-sided MSS tumors with intermediate TMB or BRAF mutation but shows limited efficacy against peritoneal or lung metastases.^169^ The number of metastatic sites alone does not predict ICI benefit.^149^

### Other predictive insights

Wnt pathway mutations correlate with reduced efficacy of ICIs combinations regardless of MSI status in mCRC.^170^ Low baseline plasma phosphocreatine identifies patients with refractory mCRC who derive greater benefit from durvalumab plus tremelimumab.^171^ In a Chinese cohort, co-mutations in DNA damage repair pathways show potential for predicting ICI response in CRC.^172^

## Conclusion

Combination immunotherapy has markedly improved outcomes for patients with dMMR/MSI-H CRC, with dual immune checkpoint inhibition emerging as a potential first-line standard in metastatic disease. Neoadjuvant immunotherapy has enabled unprecedented rates of organ preservation, and later-line regimens continue to yield durable tumor control and sustained survival benefits.

By contrast, pMMR/MSS CRC remains largely refractory to current immunotherapeutic strategies. Although combining immunotherapy with chemotherapy, targeted therapy, and/or radiotherapy has shown encouraging activity, clinical outcomes are still suboptimal, underscoring the need for more effective combinatorial approaches. Major obstacles include the scarcity of reliable predictive biomarkers, heterogeneity in metastatic sites, and an incomplete understanding of resistance mechanisms.

Future efforts should prioritize (1) the integration of multi-omics data to develop predictive models for personalized treatment selection; (2) the development of next-generation immunotherapies that enhance antitumor immunity while counteracting immunosuppressive components of the TME; and (3) the use of advanced preclinical models—such as patient-derived organoids and xenografts—to decipher mechanisms of resistance and facilitate the translation of novel strategies into clinical practice.

## Acknowledgments

Figures 1, 2, and 3 are created using BioRender.com. We are thankful to many scientists in the field whose seminal works are not cited due to space constraints. This study was supported by grants from 10.13039/501100001809National Natural Science Foundation of China (nos. 81372629, 81772627, 81874073, 81974384, 82173342, and 82403920), key projects from the Nature Science Foundation of Hunan Province (nos. 2021JJ31092, 2021JJ31048, 2024JJ6662, and 2025JJ20077), the projects from Beijing CSCO Clinical Oncology Research Foundation (nos. Y-HR2019-0182 and Y-2019Genecast-043), the Science and Technology Innovation Program of Hunan Province (no. 2024RC3042), the Youth Science Foundation of 10.13039/501100011790Xiangya Hospital (no. 2023Q01), the Postdoctoral Fellowship Program of the CPSF under grant number GZC20242044, the 10.13039/501100002858China Postdoctoral Science Foundation under grant numbers 2024M753679 and YJA20250115, and the Nature Science Foundation of Changsha (no. kq2403008).

## Author contributions

Y.H., S.Z., J.F., and C.C. had the idea for the article; J.F. and C.C. performed the literature search and finished the manuscript and figures; W.W. finished the tables; H.S., S.Z., and Y.H. made critical revisions and proofread the manuscript. All authors read and approved the final manuscript.

## Declaration of interests

The authors declare no competing interests.
